# Invasive Pasteurella multocida Infection With Cervical Abscess and Sternoclavicular Septic Arthritis Following a Cat Scratch in a Patient With Lymphedema: A Case Report

**DOI:** 10.7759/cureus.99154

**Published:** 2025-12-13

**Authors:** Sara Dourado, João Parodi, João Neves Maia

**Affiliations:** 1 Internal Medicine, Hospital Santo António, Unidade Local de Saúde Santo António (ULSSA), Porto, PRT

**Keywords:** cat scratch, pasteurella multocida, secondary lymphedema, septic arthiritis, s: metabolic syndrome

## Abstract

Pasteurella multocida is a zoonotic pathogen transmitted through animal bites or scratches. Although most infections are localized, invasive disease may occur in vulnerable patients.

An 86-year-old woman with metabolic syndrome and chronic right upper limb lymphedema developed fever and extensive inflammatory signs shortly after a superficial cat scratch. She had previously completed one course of amoxicillin with clavulanic acid and another of clindamycin without improvement. Blood cultures isolated Pasteurella multocida. Imaging revealed right upper-limb cellulitis, a cervical phlegmon, septic arthritis of the left sternoclavicular joint, a large anterior thoracic wall abscess and a posterior cervical abscess at the C4-C5 level. She received six weeks of intravenous ceftriaxone followed by six weeks of oral ciprofloxacin, with complete resolution.

Minor animal-related injuries in patients with chronic lymphedema can evolve into severe invasive *P. multocida *infections. Early recognition, prompt imaging, and prolonged targeted antimicrobial therapy are essential when surgical drainage is not feasible or is associated with unacceptable risk.

## Introduction

Pasteurella is a small Gram-negative coccobacillus, with P. multocida being the species most commonly responsible for human disease. It is a commensal organism of the oral and upper respiratory tract of many animals, particularly cats and dogs, with especially high carriage rates in cats, which explains the strong association with bite- and scratch-related infections [1]. In most cases, infection remains localized and self-limited. However, invasive disease may occur with bacteremia, deep abscesses, septic arthritis, endocarditis, or meningitis. Although rare, it typically occurs in patients with underlying comorbidities or impaired host defenses [2]. Sternoclavicular septic arthritis represents a particularly rare manifestation of P. multocida infection, accounting for a very small proportion of all cases of septic arthritis, and is usually associated with bacteremia or contiguous spread [3,4]. Likewise, deep cervical abscess formation due to P. multocida is exceptional and potentially life-threatening due to the risk of airway compromise and mediastinal extension. The simultaneous occurrence of both entities following a minor cat-related injury is exceedingly uncommon.

## Case presentation

An 86-year-old woman with metabolic syndrome and chronic right upper limb lymphedema following right mastectomy with axillary lymph node dissection for breast cancer presented with fever and rapidly progressive inflammatory signs affecting the entire right upper limb shortly after adopting a young cat. A superficial scratch caused by the cat’s claws on the distal forearm preceded symptom onset. She had no prior history of soft tissue infections in the affected limb.

Initial symptoms included fever, progressive erythema, edema, pain, and bullous skin changes involving the entire right upper limb with axillary extension. She was initially treated in primary care with amoxicillin-clavulanic acid, followed by clindamycin, without clinical improvement.

Upon admission, the patient was febrile (temperature 38.7 °C), hemodynamically stable with a blood pressure of 123/60 mmHg, heart rate of 85 beats per minute, and peripheral oxygen saturation of 96% on room air. Physical examination revealed diffuse edema and inflammatory skin changes over the entire right upper limb with cervical and right lateral thoracic extension, along with a poorly defined soft cervical swelling on the left side. No respiratory distress was evident. Laboratory evaluation revealed leukocytosis with neutrophilia, markedly elevated C-reactive protein (360 mg/L), plasma glucose of 128 mg/dL, acute kidney injury (KDIGO stage 1), and severe hyponatremia (serum sodium 120 mmol/L), and a normal serum lactate level (1.65 mmol/L). Blood cultures grew Pasteurella multocida. Antimicrobial susceptibility testing demonstrated full susceptibility to penicillin, ampicillin, amoxicillin-clavulanic acid, cefotaxime, ciprofloxacin, levofloxacin, tetracycline, and trimethoprim-sulfamethoxazole. Given the proximal extension of infection, bacteremia, systemic toxicity, and failure of outpatient antibiotic therapy, an intravenous ceftriaxone was initiated.

Initial contrast-enhanced cervical and thoracic computed tomography (CT) demonstrated a cervical phlegmon with involvement of superficial and paratracheal fat on the left side and mild cutaneous thickening of the right axilla with residual subcutaneous calcifications, without a well-defined drainable collection or mediastinal involvement. After multidisciplinary discussion, no surgical intervention was indicated, and antibiotic therapy was maintained.

**Figure 1:**
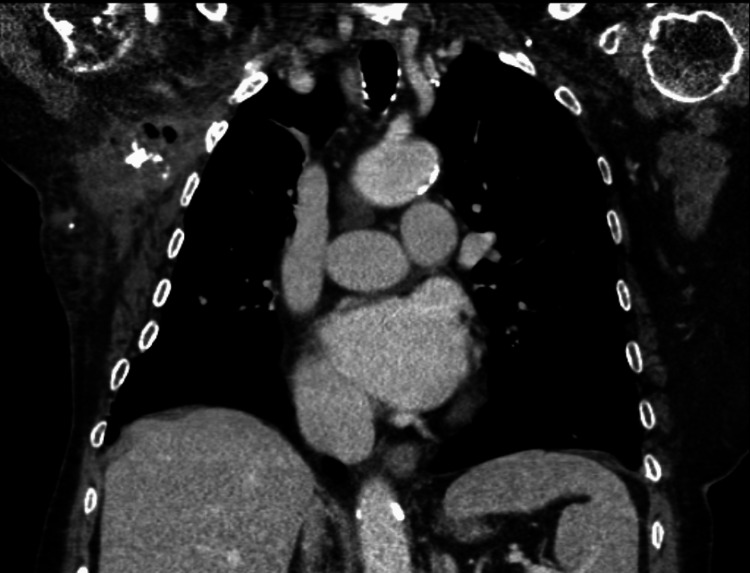
Coronal contrast-enhanced CT Coronal contrast-enhanced computed tomography showing extensive soft-tissue oedema and fat stranding of the right axilla with contiguous extension to the lower cervical and upper thoracic regions.

**Figure 2:**
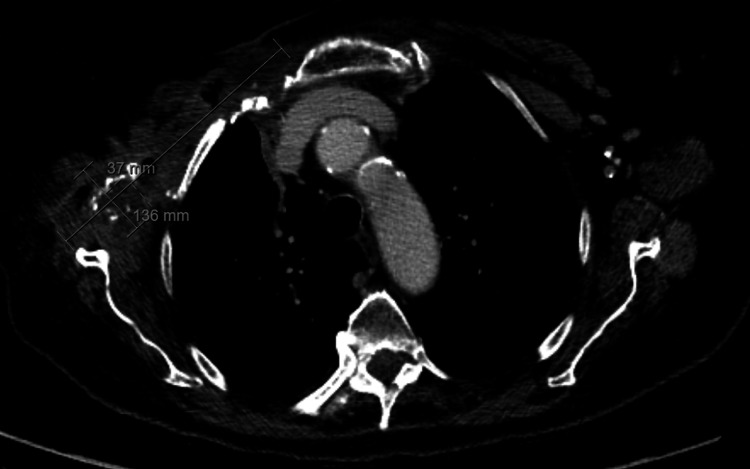
Axial computed tomography showing right anterior thoracic abscess and left sternoclavicular septic arthritis A right-sided fluid-and-gas collection measuring approximately 136 × 37 millimetres (anteroposterior × transverse) is seen in the upper anterior hemithorax, extending inferiorly toward the right axillary region, compatible with an abscess. Axial computed tomography also demonstrates features suggestive of septic arthritis of the left sternoclavicular joint, including intra-articular fluid, internal gas foci and marked subcutaneous fat stranding.

**Figure 3:**
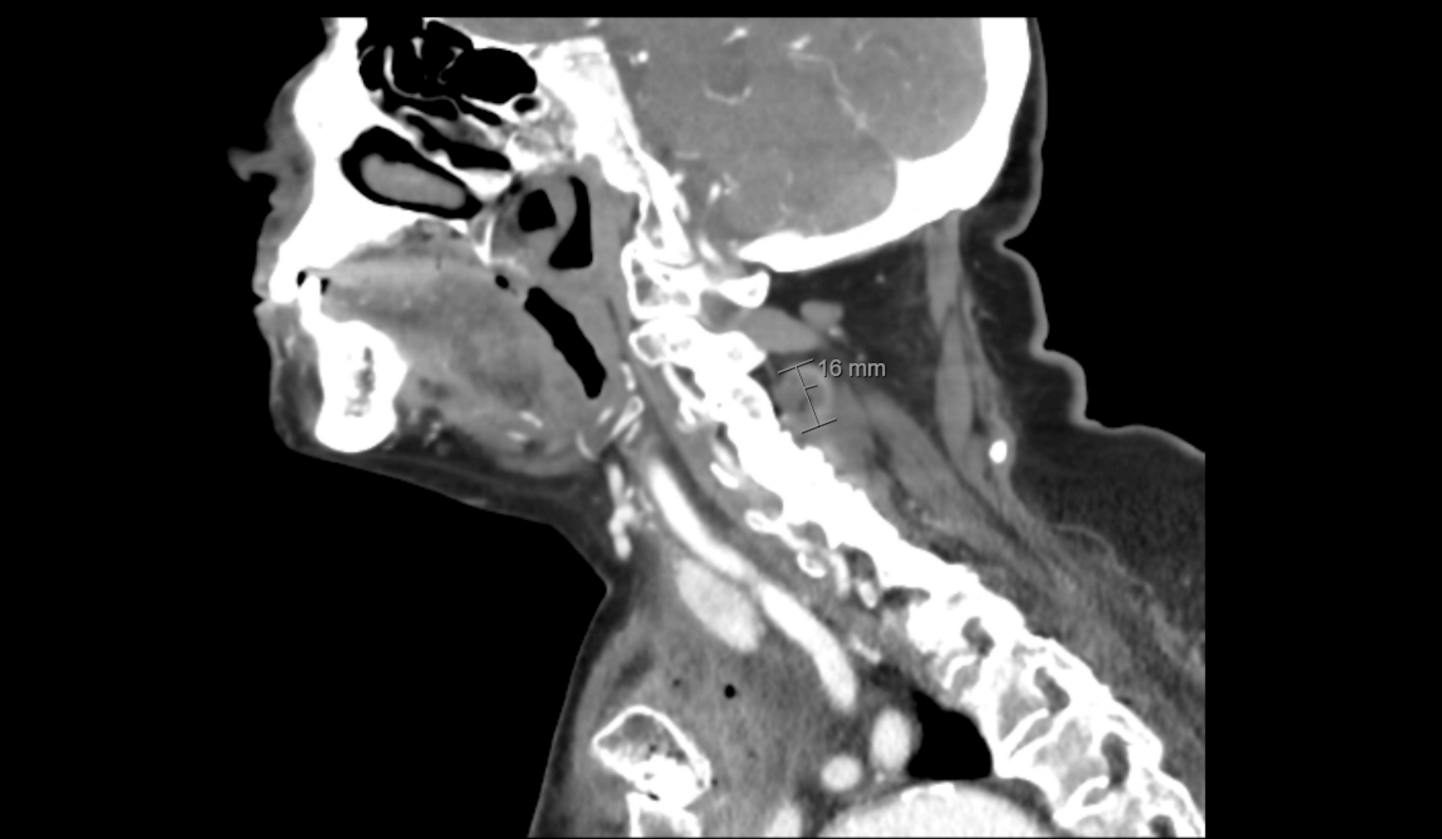
Posterior cervical paravertebral abscess at the C4–C5 level on sagittal computed tomography Sagittal computed tomography of the cervical spine demonstrating a posterior cervical paravertebral abscess at the C4–C5 level, measuring approximately 16 millimetres in depth, associated with surrounding inflammatory changes of the posterior elements.

Despite initial clinical improvement - with reduction of limb inflammation, normalization of renal function, and correction of hyponatremia - the patient remained persistently febrile. Repeat cervico-thoracic CT revealed formation of a phlegmon in the right anterior thoracic wall, signs of left sternoclavicular septic arthritis, and a small poorly defined posterior paravertebral abscess at the C4-C5 level (Figures 1, 2, 3).

The case was discussed with General Surgery and Orthopedics. Given the patient’s advanced age, frailty, radiodermatitis-related skin changes over the infected area, and absence of well-defined drainable collections, a conservative approach was maintained. Ultrasound-guided aspiration of the left sternoclavicular joint yielded purulent material, although cultures remained negative. Antibiotic therapy was temporarily escalated to piperacillin-tazobactam, with subsequent de-escalation back to ceftriaxone after repeated negative microbiological results.

Follow-up imaging after four weeks of therapy demonstrated partial regression of anterior cervical inflammatory changes and persistent posterior cervical involvement. 

The patient showed gradual clinical improvement with resolution of fever, sustained decline in inflammatory markers, and progressive reduction of cervical and thoracic edema. Infective endocarditis was excluded, including by transesophageal echocardiography. 

She completed a total of six weeks of intravenous antibiotic therapy with ceftriaxone and was transitioned to oral ciprofloxacin due to its superior bone and joint penetration, with a planned total treatment duration of approximately 12 weeks.

At five-month follow-up, the patient remained asymptomatic, afebrile, with no clinical or radiological evidence of persistent or recurrent infection.

## Discussion

Invasive Pasteurella multocida infections remain uncommon but potentially life-threatening, particularly in elderly patients and in those with metabolic disorders or impaired local or systemic immunity. Although most human infections are limited to localized skin and soft tissue involvement following animal bites or scratches, contemporary literature continues to report severe presentations involving bacteremia, septic arthritis, osteomyelitis, and deep tissue abscesses, especially in vulnerable hosts [4,5]. These reports reinforce the aggressive potential of this pathogen in predisposed patients, even after seemingly minor animal-related injuries.

In the present case, two major predisposing factors likely contributed to disease severity: chronic right upper limb lymphedema and metabolic dysfunction. Chronic lymphedema is increasingly recognized as a localized immunocompromised state. Impaired lymphatic drainage leads to reduced immune surveillance, altered antigen trafficking, and decreased bacterial clearance, thereby facilitating bacterial persistence and deep tissue spread following minor cutaneous breaches [6]. This mechanism plausibly explains the rapid progression from superficial inoculation to extensive cervicothoracic involvement observed in this patient.

Metabolic dysfunction further contributes to impaired host defense through alterations in innate immunity, particularly neutrophil chemotaxis, phagocytosis, and inflammatory signaling [7-9]. The markedly elevated C-reactive protein concentration at presentation (360 mg/L) reflects a strong systemic inflammatory response, while the normal serum lactate level and preserved hemodynamic status indicate the absence of overt septic shock. This dissociation illustrates how severe inflammatory burden may coexist with initial clinical stability in high-risk elderly patients.

Sternoclavicular septic arthritis represents a rare manifestation of P. multocida infection and is typically associated with bacteremia or contiguous spread. When present, it may lead to severe complications including osteomyelitis and chest wall abscess formation [3,4]. Deep cervical abscess caused by P. multocida is likewise exceptional and carries significant clinical risk due to the potential for airway compromise and mediastinal extension. The simultaneous occurrence of these two entities following a minor cat-related injury, as observed in this case, is exceedingly uncommon and underscores the invasive potential of this pathogen in patients with impaired local and systemic host defenses.

The negative microbiological culture obtained from the sternoclavicular joint aspirate is most plausibly explained by prior exposure to broad-spectrum antimicrobial therapy and by the timing of sampling relative to antibiotic initiation. This limitation is well recognized in the diagnostic evaluation of septic arthritis and deep tissue infections and does not exclude true infection when supported by positive blood cultures, compatible radiologic findings, and a clear clinical response to targeted antimicrobial therapy, as occurred in this patient [2].

Finally, this case highlights the critical importance of early cross-sectional imaging and prompt escalation of antimicrobial therapy in patients demonstrating rapid clinical progression or failure of initial outpatient treatment. When surgical drainage is not feasible due to patient frailty, unfavorable anatomical location, or the absence of well-defined drainable collections, prolonged intravenous antimicrobial therapy followed by an adequate course of oral consolidation remains essential for achieving complete resolution and preventing relapse.

## Conclusions

Patients with chronic lymphedema and metabolic comorbidities represent a particularly vulnerable population for severe and invasive Pasteurella multocida infections, even after apparently minor animal-related trauma. This case highlights the critical importance of early recognition, prompt cross-sectional imaging, and timely escalation to prolonged targeted antimicrobial therapy to prevent deep tissue extension and life-threatening complications. Importantly, failure of first-line oral antibiotic therapy should be regarded as a clinical red flag, mandating urgent reassessment and investigation for invasive disease.
